# Sialyllactose and Galactooligosaccharides Promote Epithelial Barrier Functioning and Distinctly Modulate Microbiota Composition and Short Chain Fatty Acid Production *In Vitro*

**DOI:** 10.3389/fimmu.2019.00094

**Published:** 2019-02-12

**Authors:** Olaf Perdijk, Peter van Baarlen, Marcela M. Fernandez-Gutierrez, Erik van den Brink, Frank H. J. Schuren, Sylvia Brugman, Huub F. J. Savelkoul, Michiel Kleerebezem, R. J. Joost van Neerven

**Affiliations:** ^1^Cell Biology and Immunology Group, Wageningen University & Research, Wageningen, Netherlands; ^2^Host-Microbe Interactomics Group, Wageningen University & Research, Wageningen, Netherlands; ^3^Microbiology and Systems Biology, The Netherlands Organization for Applied Scientific Research, Zeist, Netherlands; ^4^FrieslandCampina, Amersfoort, Netherlands

**Keywords:** epithelium, galactooligosaccharides, microbiota, short chain fatty acids, sialyllactose

## Abstract

Human milk oligosaccharides (HMO) and prebiotic oligosaccharides are proposed to confer several health benefits to the infant. They shape the microbiota, have anti-inflammatory properties, and support epithelial barrier functioning. However, in order to select the best oligosaccharides for inclusion in infant formulas, there is a need to increase our understanding of the specific effects of HMO and prebiotics on the host immune system. Therefore, we investigated the effects of the HMO sialyllactose (SL), and galactooligosaccharides (GOS) on epithelial barrier functioning, microbiota composition, and SCFA production. The effect of GOS and SL on epithelial barrier functioning and microbiota composition was investigated using *in vitro* models. Epithelial barrier function was investigated by transcriptome analysis of fully polarized Caco-2 cells exposed for 6 h to SL or GOS. In addition, epithelial cell growth, alkaline phosphatase production, and re-epithelization was studied. Further, we investigated the effect of SL and GOS on microbiota composition and SCFA production using *in vitro* fecal batch cultures. Transcriptome analysis showed that SL and GOS both induced pathways that regulate cell cycle control. This gene-expression profile translated to a phenotype of halted proliferation and included the induction of alkaline phosphatase activity, a marker of epithelial cell differentiation. SL and GOS also promoted re-epithelialization in an *in vitro* epithelial wound repair assay. SL and GOS did show distinct modulation of microbiota composition, promoting the outgrowth of *Bacteroides* and bifidobacteria, respectively, which resulted in distinct changes in SCFA production profiles. Our results show that SL and GOS can both modulate epithelial barrier function by inducing differentiation and epithelial wound repair, but differentially promote the growth of specific genera in the microbiota, which is associated with differential changes in SCFA profiles.

## Introduction

Microbial colonization directly after birth and breastfeeding are crucial events that determine health in neonatal and adult life (1, 2). Breast milk is a unique mixture of immunoregulatory proteins, antimicrobial peptides, micronutrients, milk fat globular membrane, miRNA containing extracellular vesicles, and human milk oligosaccharides (HMO) (3, 4). It has been widely recognized that breast milk confers health benefits to the infant by shaping the microbiota, preventing infections and promoting cognitive abilities (5). Breast milk serves as the golden standard for infant nutrition and exclusive breastfeeding for the first 6 months in life is advocated by the WHO (6). Nevertheless, more than half of the infants is not exclusively breast-fed during the first 6 months of life worldwide (5) and is thus dependent on infant nutrition. At present, infant formulas are commonly supplemented with prebiotic oligosaccharides like galactooligosaccharides (GOS), fructooligosaccharides (lcFOS), or polydextrose, or a mixture of these. Among these polymeric glycans, GOS is the most widely used prebiotic oligosaccharide in infant nutrition.

Contrary to other mammals, human breast milk contains a very high amount and a structurally diverse set of oligosaccharides that even exceeds the protein content of breast milk (7–9). HMO consist of a lactose backbone that can be elongated into polymeric glycan structures that can contain fucose or sialic acid moieties. The simple trisaccharides 3′-sialyllactose (3′SL) and 6′-sialyllactose (6′SL) are among the most abundant sialylated HMO, with estimated concentrations in mature breast milk ranging between 170–500 μg/ml 6′SL and 76–300 μg/ml for 3′SL (10). Compared to human milk, mature bovine milk contains low levels of 3′SL (30–119 μg/ml) and 6′SL (17–88 μg/ml) (10).

Exogenous sialic acids have been suggested to be essential for brain development in early life (11). Additionally, sialylated oligosaccharides may, although mostly investigated *in vitro* and animal models, exert other health effect via neutralization of pathogens, fermentation into anti-inflammatory metabolites, direct immunomodulation, and improved epithelial barrier functioning (10). HMO and prebiotics are metabolized by the microbiota in the colon and upon their colonic fermentation, sialylated oligosaccharides may function as an essential exogenous source of sialic acid. Animal models have shown that endogenous production of sialic acid in the liver is low during the first weeks of life (12), suggesting that microbial production of sialic acid in neonates could be relevant, and emphasizing the importance of proper colonic microbial colonization and fermentation.

It has been suggested that growth factors in human milk are important for epithelial barrier functioning. For instance, breast milk contains epidermal growth factors that promote the proliferation and differentiation of epithelial cells, thereby promoting gut maturation (13, 14). Improving gut maturation by means of nutrition in early life may be particularly relevant in the first weeks of life, for premature infants and infants suffering from intestinal infections or inflammatory bowel conditions (15, 16). Furthermore, nutrition may play a role in maintaining barrier function later in infancy during adverse episodes, e.g., when the epithelial layer is challenged by inflammation due to infection or physical damage.

The neonatal microbiota is shaped by colonization of bacteria from the mother during vaginal delivery and is further shaped by breastfeeding (17). As reviewed by Mueller et al. (19), introduction of infant formula instead of breastfeeding results in microbial changes. Breastfed children show a lower microbial diversity, decreased abundance of Clostridiales and Bacteroidetes members (18), a lower prevalence of *C. difficile* and *E. coli* and increased prevalence of bifidobacteria (17, 19, 20) and lactobacilli (17, 21). The capacity of bifidobacteria to digest HMO has been appreciated since the 1950s, which is the most likely explanation of the expansion of this bacterial group in breastfed infants.

The best described and most abundant group of microbial-derived metabolites are short-chain fatty acids (SCFA). SCFA such as acetate, propionate, and butyrate are produced in the colon and reach high concentrations (20–100 mM) locally and much lower levels systemically (in the μM range) (22). The increase in bifidobacteria was correlated with an increase in fecal lactate and acetate concentrations and lower pH (23). SCFA are shown to exert direct anti-inflammatory effects on colonocytes and restore epithelial barrier functioning, which results in suppression of colitis in animal models (24, 25). Additionally, SCFA may exert systemic responses via inducing epigenetic changes and directly modulating gene transcription in immune cells. For instance acetate was shown to enhance regulatory T cell functioning by epigenetic modification of the FOXP3 gene (26). This study by Thorburn et al. (26) and comparable studies in other mouse models show that the diet can, via microbial-derived SCFA, alleviate respiratory diseases (26–28). Although largely unexplored, fermentation of HMO, or prebiotics in the colon may thereby serve as an important carbohydrate source that may impact the physiology and health of the infant (29–31).

To introduce part of the functionality of HMO into infant nutrition, prebiotic oligosaccharides like GOS have been added to infant formulas to support the outgrowth of bifidobacteria. Prebiotics have no detrimental effect on growth of the infant and may reduce the pH and increase the softness of the stool, increase stool frequency, and increase the fecal *Bifidobacterium* and *Lactobacillus* counts (32–36). Fecal SCFA levels of neonates fed infant formula supplemented with a 9:1 GOS:FOS mixture are more similar to breast-fed neonates compared to infants that are bottle-fed without prebiotic supplementation (37). Sialyllactose (SL) can be extracted from bovine milk, which could be of interest for early life nutrition (38). However, a better mechanistic understanding of individual HMO and currently used prebiotics is required. SL and GOS may shape microbiota composition and epithelial barrier functioning, since they are only partly metabolized in the GI tract (39, 40). The aim of our study was therefore to investigate the effects of SL and GOS on epithelial barrier function (i.e., re-epithelialization, proliferation, and differentiation of Caco-2 cells; supported by underlying mechanisms using transcriptomics), and their effects on microbiota composition and subsequent SCFA production. To this aim, we applied multiple *in vitro* models using human epithelial cell lines and adult and infant fecal batch culture experiments.

## Materials and Methods

### Sialyllactose and Galactooligosaccharides

SL (FrieslandCampina) was isolated from bovine milk and used for gene-expression profiling on Caco-2 cells and batch culture experiments. The phenotypic effect of SL and GOS on Caco-2 and Ca9-22 cells was confirmed using mixtures of chemically synthesized 3′SL (OS04397, Carbosynth) and 6′SL (OS04398, Carbosynth). Moreover, concentrations as found in cow's milk (25 μg/ml 3′SL + 75 μg/ml 6′SL), human milk (125 μg/ml 3′SL + 375 μg/ml 6′SL) or human colostrum (750 μg/ml 3′SL + 2,250 μg/ml 6′SL) were included (10). Higher concentrations (5 and 10 mg/ml) of equal amounts of 3′SL and 6′SL were also included in all cellular assays using Caco-2 cells as they match the concentration used for transcriptome profiling. The concentration of GOS (FrieslandCampina) was matched to the total concentration of SL.

### Cell Culturing

Ca9-22 (JCRB0625) gingival epithelial cells and Caco-2 cells (HTB-37) colon epithelial cell lines were purchased from the National Institute of Biomedical Innovation JCRB Cell Bank (Osaka, JP) and ATCC (Manassas, USA), respectively. Both cell lines were cultured in Dulbecco's Modified Eagle Medium (DMEM) containing Glutamax (Gibco, Invitrogen, Paisley, UK) and 10% fetal calf serum (FCS), 100 U/ml penicillin and 100 μg/ml streptomycin (Sigma-Aldrich, MO, USA). The cell lines were cultured at 37°C in a humidified atmosphere containing 5% CO_2_ and passaged every second day. Experiments were performed using cell-passage numbers 9–20 and 12–41 for Ca9-22 and Caco-2, respectively.

### Caco-2 Stimulation and RNA Extraction for Microarray Analysis

One million cells were seeded and polarized for 2 weeks in each well of a 6-wells Transwell system for microarray analysis. The polarized cell layer was incubated with 10 mg/ml sialyllactose isolated from bovine milk (FrieslandCampina) or GOS (FrieslandCampina). Medium was refreshed every second day. All treatments and controls were performed in triplicate. Medium was aspirated from the inserts and TRIzol was added to lyse the cells. RNA was extracted by first adding 200 μl of chloroform to 1 ml of the thawed cell lysate. Next, samples were vortexed for 15 s, incubated for 2–3 min at room temperature and centrifuged for 15 min at 11,904 g at 4°C. The upper aqueous phase was transferred to a new tube and 500 μl isopropanol was mixed with the sample and incubated for 5–10 min at room temperature to precipitate the RNA. The samples were then centrifuged for 10 min at 11.904 g at 4°C. Supernatants were discarded and the pellet was washed with 1 ml 75% ethanol and centrifuged for 10 min at 4°C. The supernatant was discarded and the pellet was dried for 5 min. 100 μl MQ was added and RNA was further purified using RNeasy Mini Kit, according to the manufacturer's recommendations (ref 74106, Qiagen).

### Gene Expression Profiling

RNA quantity and quality was assessed spectrophotometrically (Nanodrop) and with 6000 Nano chips via a Bioanalyzer 2100 device (Agilent, Santa Clara, CA, USA), respectively. RNA was judged as being suitable for array hybridization only if samples showed intact bands corresponding to the 18S and 28S ribosomal RNA subunits, displayed no chromosomal peaks or RNA degradation products, and had a RIN (RNA integrity number) above 8.0. The Ambion WT Expression kit (Life Technologies, cat. no. 4411974) in conjunction with the Affymetrix GeneChip WT Terminal Labeling kit (Affymetrix, Santa Clara, CA; cat. no. 900671) was used for the preparation of labeled cDNA from 100 ng of total RNA without rRNA reduction. Labeled samples were hybridized on Affymetrix GeneChip Human Gene 1.1 ST arrays that contain 30,000 coding transcripts and over 11,000 long intergenic non-coding transcripts, provided in plate format. Hybridization, washing, and scanning of the array plates was performed on an Affymetrix GeneTitan Instrument, according to the manufacturer's recommendations. Quality control of the hybridizations to the Human Gene 1.1 ST array and primary data analysis were performed according to strict criteria to ensure that the array data were of the highest possible quality.

### Statistical and Functional Analysis of Microarray Data

Packages from the Bioconductor project (41) integrated in the on-line MADMAX pipeline (42) were used for analyzing the scanned Affymetrix arrays including quantile normalization and expression estimates via Robust Multiarray Analysis (RMA) and empirical Bayes approach using the Bioconductor library affyPLM. For array annotations, we used the following software and database versions: R-version 2.11.1, Bioconductor version 2.6; Custom CDF (remapped CDF v13, November 2010, library: hugene11stv1hsentrezgcdf, and hugene11stv1hsentrezg.db version 13.0.0; http://brainarray.mbni.med.umich.edu). Gene functional annotations, gene ontology (GO) enrichment and differential expression calculations, biological interpretation of transcriptome datasets and pathway analysis were carried out using Bioconductor packages and third-party software modules and Ingenuity Pathway Analysis (IPA) (Qiagen), following the approach described in Baarlen et al. (43). Various quality metrics, diagnostic plots, pseudo-images and classification methods were applied to ascertain that only arrays that passed the most rigorous quality controls (44) were used in the subsequent analyses using the criteria described in Baarlen et al. (43). Here, probe sets were redefined according to Dai et al. (45) based on the Entrez Gene database, and differentially expressed probe sets were identified using Bioconductor's limma package (46); limma values were compared to a moderated T-statistic (IBMT) (47) when differential gene expression according to limma displayed low fold-changes and *P*-values were just below significance cut-offs. P-values were corrected for multiple testing using a false discovery rate (FDR) method (48). FDR values between *p* < 10^−7^ and *p* < 10^−8^ were chosen so that for all comparisons, the number of genes included in pathway analyses were about 800 genes.

### Verification of Transcriptome Results by qPCR

One million Caco-2 cells were polarized for 2 weeks on flat bottom plates and stimulated for 6 h with 10 mg/ml sialyllactose (5 mg/ml 3′SL + 5 mg/ml 6′SL) for verification of the transcriptome analysis by quantitative PCR (qPCR analysis). Medium was aspirated and cells were lysed in RNAeasy Lysis buffer. RNA was 1:1 diluted with 70% ethanol and homogenized using a syringe. A RNeasy Mini Kit was used according to the manufacturer's recommendations to isolate RNA for qPCRs (ref 74106, Qiagen). Cells were stored at −80°C until further use. Next, 1 μg of RNA was treated with 1U DNase (Qiagen, Germany) for 15 min at room temperature. DNase activity was inhibited with 25 mM EDTA (Invitrogen) followed by incubation for 10 min at 65°C. 300 ng of random primers and 0.5 mM dNTPs were added and incubated for 10 min at 65°C followed by 5 min at 4°C for annealing. Thereafter a mixture of 200 U of Superscript III (Invitrogen), 0.1M DTT (Invitrogen) and 40 of RNAse OUT inhibitor in first strand buffer was incubated for 10 min at 21°C. The reaction was performed for 50 min at 50°C using a Biometra T3 Thermocycler (Westburg, The Netherlands). The reverse transcriptase reaction was inactivated by incubation at 70°C for 15 min. cDNA was stored at −20°C. A mixture containing 7 μl SYBR Green Mix, 1 μg cDNA template and 2.1 μM of forward and reverse primers per sample was run on the Rotor Gene Real-timer Cycler (Corbett Research, the Netherlands). Genes were selected based on the microarray data according to the following criteria: (1) raw expression values should exceed 20 in treatment or control and exceed 100 in the opposite part of the comparison; (2) limma fold-changes should be as large as possible and at least exceed (-)2 in log2-space; (3) FDR *p*-values should be as low as possible; and (4) genes fulfilling these criteria should not be exclusively involved in responding to salt exposure or ion transport across membranes (salts might be common contaminants of SL preparations). We reasoned that the criteria above would enable us to select genes of which the expression was sufficiently modulated upon exposure to SL to be measurable by qPCR and sufficiently relevant to compare SL quality of different batches. Primers amplifying genes that were highly modulated by SL in the microarray (Table 1) were designed by the authors and synthesized by BaseClear. In total, 35 reaction cycles consisting of 95°C for 15 s, 60°C for 30 s, and 72°C for 45 s were run followed by a final hold of 60°C for 1 min. Data was analyzed using RotorGene Q series sofware (Qiagen). Relative gene-expression was calculated using the Pfaffl method (49).

**Table 1:** List of qPCR primers for verification of commercial SL.

		**Primer sequence**
FOXM1	FW	GCCTATCCAACATCCAGTG
	RV	CCGCTCAGACACAGAGTT
IFIT1	FW	GTGTCCAGAAATAGACTGTGA
	RV	CCATCCAGGCGATAGGCA
CDK1	FW	TCAACTCTTCAGGATTTTCAG
	RV	GGATGATTCAGTGCCATTT
EDNRB	FW	CTTGGCTCTGGGAGACC
	RV	CACGGAGGCTTTCTGTAT
MARCH3	FW	CTGTCGCACTCTTCACTATTTA
	RV	CAGACTTTGGAATGAGGAGAATC
TNFRSF9	FW	GTAAACAAGGTCAAGAACTGA
	RV	CCATTCACAAGCACAGAC
GAPDH	FW	TGCACCACCAACTGCTTAGC
	RV	GGCATGGACTGTGGTCATGAG

### Caco-2 Cell Counts and Alkaline Phosphatase Measurements

Caco-2 cells were seeded at a density of 200.000 cells/well in flat bottom 24-wells plates. GOS or SL were added to the wells for 4 days. The supernatant was collected and quantified for secreted alkaline phosphatase by QUANTI-Blue (Invivogen, rep-qb2) as a marker of differentiation. QUANTI-Blue powder was reconstituted according to the manufacturer's instructions. 60 μl supernatant was added to 190 μl QUANTI-Blue and incubated for 3 h at 37°C. The optical density (OD) at 625 μm was measured using a FilerMAX F5 (Molecular Devices, Nederman, Germany). To count the cells, trypsin was added to the cells. The cells were spun down at 300 g for 5 min and washed twice with PBS. 150 μl Trypsin-EDTA (0.25%) (ThermoFisher, 25200) was added to detach the cell monolayer. Trypsin was inactivated by resuspending the cells in 150 μl PBS + 4% FCS + 0.02% EDTA. Dead cells were stained with DRAQ7 (Abcam; ab109202) and 50 μl of 0.975E6 beads/ml (Fluoresbrite YG Carboxylate microspheres 10 μm, 18142) were added to count cells (Figure S2A; gating strategy). Cells were acquired on a BD FACS Canto II (BD Biosciences) and analyzed using the FlowJo software V10.

### Scratch Assay and Automated Image Acquisition and Segmentation

To investigate the effect of SL and GOS on re-epithelialization, a scratch assay and analysis was performed as previously described (50). In short, ~35,000 cells/well were seeded in 96-wells plates in DMEM + 10% FCS and grown overnight into a confluent monolayer. The next day, cells were starved for 2 h by replacing the medium with DMEM without FCS. The cellular cytoplasm was labeled with 2 μM CellTracker™ Red CMTPX (Molecular Probes, OR, USA) and the nuclei were stained with 2 μg/ml Hoechst 33342 (Molecular Probes, OR, USA). Longitudinal scratches (0.3 × 2 mm) were introduced in the monolayers using the HTSScratcher (Peira, Antwerpen, BE). After washing away the cell debris, 100 μl of a mixture of 3′SL and 6′SL or matching concentrations of GOS, 4 ng/ml TGFα (R&D Systems, MN, USA) or 10 μM of p38 inhibitor (SB203580; Cell Signaling Technology, MA, USA) +10 μM MEK1/2 inhibitor (U0126, Cell Signaling Technology) in DMEM without FCS were added into the wells in a randomized manner. The BD Pathway 855 Bioimaging System (BD Biosciences, CA, USA) was programmed to acquire fluorescent and bright-field images every 20 min for 5 h. Image segmentation was performed using CellProfiler 2.1.1 and visualized in FCS Express 4 Plus (*De Novo* Software, CA, USA) software. The lag time (λ in min), repair rate (μ_m_ in cells minute^−1^) and the maximum number of cells (A) within the scratch area of each well were calculated in R by fitting the modified Gompertz equation through the re-epithelialization measurements.

### Fecal Sample Preparation and Batch Cultures

A pooled preparation of infant or adult fecal samples was prepared to test the prebiotic effect of SL and GOS in a batch culture system. All participants had no known gastrointestinal disorders and did not consume prebiotic products 3 months prior to sampling. Infant feces was collected from 13 infants from 1 to 6 months of age who did not receive solid food, antibiotics, prebiotics or probiotics in the last month. 6 g per infants was pooled in 300 ml dilution medium containing lactose (1% w/v) as a carbon source as a replacement for the carbohydrates (e.g., starch, pectin) used in adult fecal cultures (51). Adult fecal slurry was prepared from fecal samples collected from 16 adult volunteers. Individual fecal samples were diluted to a 10% w/v mixture using 0.1M, pH7, reduced PBS and pooled. This fecal slurry was homogenized for 120 s at normal speed (Seward Stomacher 80 Biomaster), aliquoted and frozen until further use. Culture medium was reduced overnight in an anaerobic cabinet (10% H2, 10% CO_2_, 80%N2) and 10% w/v fecal slurry was added the next day. Cultures were kept at 37°C for 24 h. Fecal slurry was collected at 3, 6, 9, and 24 h post-inoculation with SL or GOS. Autoclaved anaerobic chemostat nutrient medium, according to the preparation described in patent WO2011096809A1 (52) was used. The chemostat vessels were equipped with magnetic stirrers and pH meters that were set to maintain pH between pH6.8 and pH7 by adding 0.5 M NaOH or 0.5HCl. 10 g/L of GOS or SL were added to the vessels just before adding 10% w/v of the fecal slurry.

### Detection of SCFA by HPLC

SCFA levels were measured in fecal samples from the batch cultures using a high pressure liquid chromatography (HPLC) as developed and described by Guerrant et al. (53).

### Quantification of Bacteria Using 16S rDNA qPCR

To assess the abundance of bacterial genera from the different batch cultures by qPCR, fecal slurry was thawed and DNA was isolated using DNA Stool Mini Kit (Qiagen) according to the manufacturer's recommendations. The differential bacterial counts were quantified using quantitative PCR. The bacterial count was calculated based on a dilution series of DNA that was isolated from bacterial strains with predetermined CFU. Primers and probes were based on 16S rDNA gene sequences retrieved by http://greengenes.lbl.gov. Primer Express software was used to design the primers.

### Intestinal Microbiota Chip

Microbiota samples from infant and adult incubations were analyzed by microarray analysis according to Ladirat et al. (54). The microarray contains over 400 bacterial primers for intestinal bacteria species (e.g., *Bacteroides, Bifidobacterium, Enterobacteriaceae, Clostridia*, and *Lactobacillus*) were selected based on literature and sequence databases. The array includes primers on different levels up to species level. Adult samples were analyzed with the I-chip which was based on adult microbiota composition, whereas the II-chip was used for analyzing the infant samples. The II-chip was based on infant microbiota composition and therefore partly includes different micro-organisms as compared to the I-chip, but all experimental protocols for working with both chips are identical (54).

### Statistics

Normality was tested with a Kolomogorov-Smirnov and Shapiro-Wilk test. A Kruskal-Wallis test with Dunnett's t (2-sided) *post-hoc* test was used to compare the GOS or SL treated cells compared to medium control. For the statistical analysis of alkaline phosphatase activity, a one-way ANOVA with Dunnett's t (2-sided) *post-hoc* test was used, although the sample size of four was too small to test for normal distribution. Significant differences were indicated by: ^***^ = *P* < 0.001, ^**^ = *P* < 0.01, and ^*^ = *P* < 0.05. IBM SPSS Statistics V23.0 was used for the statistical analysis.

## Results

### SL and GOS Modulate Cell Cycle Control and Induce Differentiation of Epithelial Cells

**Figure 1:**
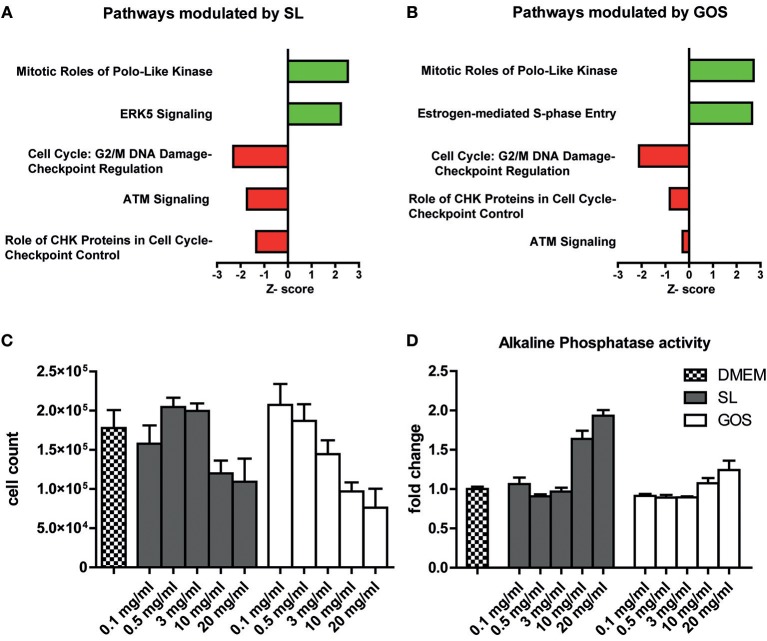
SL and GOS modulate cell proliferation and induce differentiation. A fully polarized epithelial layer of Caco-2 cells cultured in DMEM +10% FCS was exposed to 10 mg/ml SL or GOS for 6 h. After 6 h the cells were lysed and RNA was isolated for microarray analysis and subsequent IPA analysis. The top five most significantly, excluding ERK5 (*p* = 0.07), regulated pathways by **(A)** SL and **(B)** GOS (IPA analysis results) were shown. Next, phenotypic changes were assessed by culturing Caco-2 cells for 4 days in the presence of different concentrations of GOS and SL (0.1–20 mg/ml), followed by **(C)** cell counting by flow cytometry (*n* = 2) and **(D)** alkaline phosphatase activity determination in culture supernatants (*n* = 4). Data was represented as mean ± SEM.

Since the majority of oligosaccharides are considered to be digested in the large intestine, undigested prebiotics may directly affect epithelial cells in the proximal regions of the intestinal tract. To investigate the effect of GOS and SL on barrier functioning, a fully polarized monolayer of Caco-2 cells that has the biochemical characteristics of a small intestinal epithelial cell line (55) was exposed for 6 h to 10 mg/ml SL or GOS. We performed a microarray to investigate what pathways were modulated by GOS or SL. IPA identified cellular pathways in Caco-2 cells that had been differentially modulated by the oligosaccharides compared to the medium control. In total, 28 pathways were significantly modulated by SL, of which 3 were predicted to be repressed and one to be induced (Table S1). Of the 25 other significantly modulated pathways, the gene expression profiles did not enable IPA to predict whether a modulated pathway was induced or repressed. Cell cycle: G2/M damage checkpoint regulation, ATM signaling and role of CHK proteins in cell cycle checkpoint control were all predicted to be downregulated (Figure 1A). The mitotic role of polo-like kinases and ERK5 were predicted to be upregulated by SL (Figure 1A).

Next, we investigated the effect of GOS on epithelial barrier transcriptomes. GOS induced the modulation of 63 pathways of which 8 were predicted to be activated and 13 to be repressed according to IPA (Table S1). Interestingly, 4 out of the top 5 most significantly modulated pathways by SL compared to medium control were also significantly modulated in the same direction by GOS (Figure 1B). Additionally, GOS was predicted to induce estrogen-mediated S phase entry and to modulate ATM signaling and role of CHK proteins, pathways that participate in DNA replication checkpoints, among others. Additionally, GOS repressed cell cycle: G2/M DNA damage checkpoint regulation and strongly induction of mitotic roles of polo-like kinases modulation. Taken together, IPA analysis showed that both oligosaccharides modulate pathways involved in cell cycle progression and mitosis (Supplementary Text and Table S2). Since the SL (FC) used for the transcriptome studies on Caco-2 was isolated from bovine milk and may contain salts, we verified the transcriptome data with qPCR using commercially available, synthesized SL (Carbosynth) (Figure S1). To investigate whether these transcriptional effects were also translated to a cell cycle progression and (or) cell proliferation phenotype, we cultured Caco-2 cells for 4 days in the presence of SL or GOS in complete medium. The number of cells and the fraction of live cells was assessed using flow cytometry (Figure S2A). The cell number was more than doubled and the monolayer was still non-confluent, suggesting little contact-inhibition (Figure S2B). Caco-2 cells grown for 4 days in the presence of high concentrations (10 mg/ml or 20 mg/ml) of SL or GOS showed a lower cell count (Figure 1C), which could partially be explained by cell death (Figure S2C). Nevertheless, GOS showed more cell death in higher concentrations (>3 mg/ml) as compared to SL (Figure S2C). This decrease in cell counts was accompanied by an increase in alkaline phosphatase activity at high concentrations of GOS and SL (Figure 1D), which suggests that SL and GOS modulate the balance between cell proliferation and cell differentiation and may thus influence intestinal homeostasis.

### SL and GOS Induce Re-epithelialization

**Figure 2:**
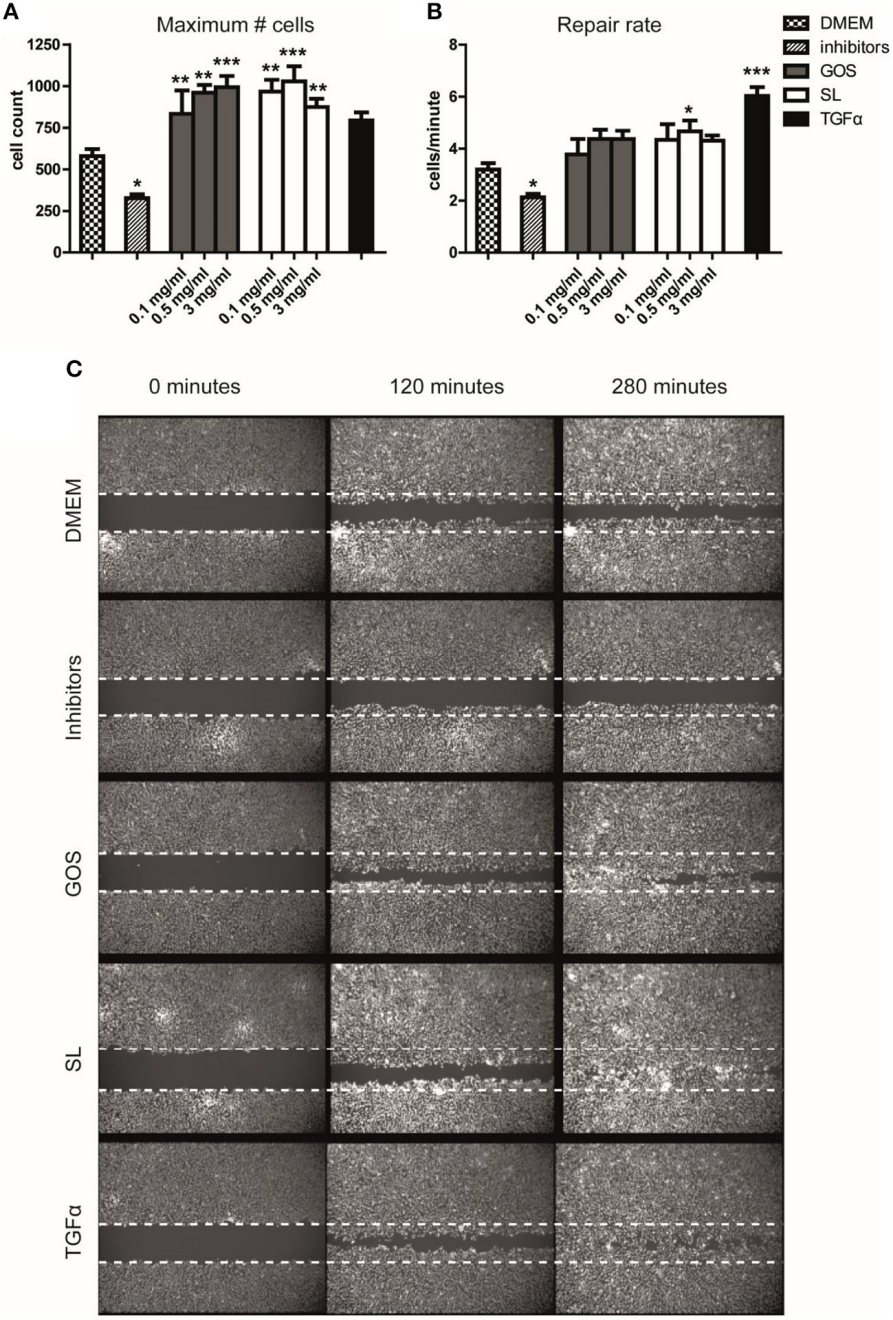
SL and GOS promote re-epithelialization, one aspect of wound healing. Longitudinal scratches were applied to a confluent layer of labeled Ca9-22 epithelial cells. The medium with cell debris was replaced by fresh medium containing mixtures of 3′SL and 6′SL as present in cow's milk (0.1 mg/ml), human milk (0.5 mg/ml) and human colostrum (3 mg/ml) or matching concentrations of GOS or inhibitors or TGFα that were used as negative and positive controls, respectively. **(A)** The total number of cells at the end of the measurement (*t* = 280min) and **(B)** the increase in cell numbers over time (i.e., repair rate) was calculated using a non-linear model using the Gompertz equation. Data of three independent experiments (*n* = 7–11) was represented as mean ± SEM. **(C)** An image of one representative well was shown (0.5 mg/ml SL or GOS). Significant differences compared to DMEM control were indicated by ^*^P < 0.05; ^**^P < 0.01 and ^***^P < 0.001.

We thus showed that GOS and SL alter cell cycle progression and, at higher concentrations, halt proliferation and induce differentiation of Caco-2 cells. We therefore investigated the effect of SL and GOS on re-epithelialization, an important phase in wound-healing where control of epithelial cell proliferation and differentiation is tightly controlled by intrinsic pathways. We used a scratch assay that tracks the presence (in terms of numbers and location) of epithelial cells in the scratch area over time. Caco-2 cells are tightly connected to neighboring cells, which makes this cell line unsuitable for scratch assays. We therefore used Ca9-22 cells (immortalized gingival-) epithelial cells (50). We tested a mixture of SL as present in cow's milk (0.1 mg/ml), human breast milk (0.5 mg/ml), and human colostrum (3 mg/ml) and the same concentrations of GOS. Strikingly, GOS and SL induced a significant increase in the maximum number of cells present in the scratch area 5 h after applying the oligosaccharides when compared to medium control (Figure 2A). The repair rate, as a non-linear measurement of cellular influx over time, also showed an increase at the lowest dose used although not significantly different when compared to the medium control (Figure 2B). The positive control TGFα, stimulated a significant increase in the repair rate (Figure 2B) whereas the maximum number of cells present in the scratch area was not significantly higher (Figure 2A). The negative control consisting of p38 mitogen-activated protein kinase- and MEK1/2 phosphorylation inhibitors resulted in a significantly lower maximum number of cells present in the scratch area and lower repair rate (Figures 2A,B). Thus, SL and GOS in concentrations present in cow's milk and breast milk both stimulated re-epithelialization of Ca9-22 in terms of a more complete closure of the scratch area within 5 h after applying the oligosaccharide mixture (Figure 2C).

### SL and GOS Distinctly Modulate Microbiota Composition

**Figure 3:**
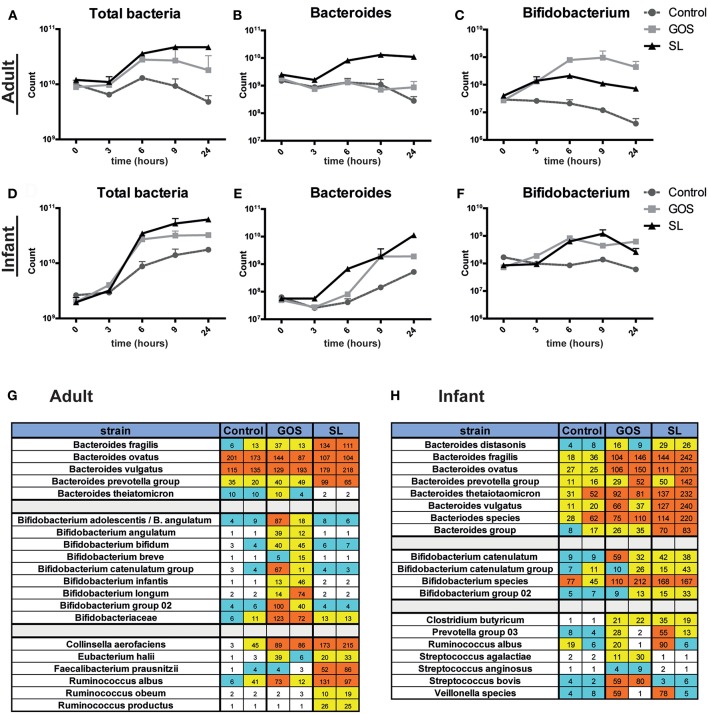
SL and GOS differentially modulate microbiota composition. Batch cultures of adult and infant pooled fecal samples cultured in growth medium were supplemented with or without SL or GOS in duplo. Fecal samples were collected at the start of the batch culture and after 3, 6, 9, and 24 h. Microbiota composition on genus level **(A–F)** and on species level **(G,H)** was determined by qPCR and chip analysis, respectively. Bacterial numbers were shown as mean ± SEM of two independent batch cultures. Raw fluorescence data are shown for both individual runs for chip analysis.

The microbiota plays an important role in gut homeostasis and barrier functioning. We therefore investigated the effects of SL and GOS on microbiota composition. Pooled adult or infant fecal cultures were spiked with 10 mg/ml SL or GOS. Fecal slurry was collected after 3, 6, 9, and 24 h for microbiota analysis. Microbiota analysis was first performed on genus level by performing qPCRs using generic or specific 16S rRNA primers to detect the numbers of total bacteria, *Bifidobacterium, Bacteroides, Lactobacillus*, and *Escherichia coli*. The starting amount of bacteria in the fecal slurry of the pooled adult samples (Figure 3A) was higher compared to those of infants (Figure 3D). Nevertheless, the total bacterial numbers in infant and adult cultures increased in the first 6 h of the culture, independent of the inoculum (Figures 3A,D). Notably, infants showed relatively to the low bacteria numbers a high amount of bifidobacteria compared to adults. In infant batch cultures, SL and GOS both induced an increase in bifidobacteria (Figure 3F) while lactobacilli numbers were not markedly increased compared to control (Figures S3A,D). Also in infant batch cultures, the number of *Bacteroides* bacteria had increased after spiking by GOS (Figure 3E). After spiking of fecal batch cultures of adults by SL, the numbers of *Bacteroides* bacteria had increased; this increase had not been induced by GOS (Figure 3B). However, again in the adult fecal batch cultures, GOS substantially promoted the outgrowth of bifidobacteria (Figure 3C). No differences were observed in the numbers of *Clostridium* and *Escherichia* (Figure S3).

Next, fecal slurries from time point 0 and 24 h were analyzed at species level using an intestinal microflora chip (I/II-chip; TNO). Of the 400 species quantified on the chip, changes in bifidobacteria, *Bacteroides* and several other species were markedly changed by the prebiotic supplements. Overall, the chip analysis confirmed the 16S rRNA qPCR data, showing that *Bacteroides* and bifidobacteria were markedly increased in adult batch cultures in the presence of SL and GOS, respectively (Figure 3G), and that this distinct pattern was less pronounced in infant fecal batch cultures (Figure 3F). In adult batch cultures, SL induced a specific increase of *Faecalibacterium prausnitzii, Ruminococcus obeum, Collinsella aerofaciens, Eubacterium halii*, and *Ruminococcus productus* (syn. *Blautia producta*, comp. nov.) (Figure 3G). GOS increased the abundance of several *Bifidobacterium* species, including *B. bifidum, B. infantis*, and *B. longum* in adult cultures (Figure 3G). In infant cultures, SL induced the specific outgrowth of members from the *B. prevotella* group and *B. thetaiotaomicron* (Figure 3H). In line with the qPCR data, this increase in abundance of *Bacteroides* species (e.g., *B. fragilis, B. thetaiomicron, B. ovatus*) induced by SL was also seen in infant batch cultures were supplemented with GOS (Figure 3H). The specific outgrowth of *Bifidobacterium* species by GOS was less pronounced in batch cultures from infants compared to adults (Figure 3H). Thus, we showed that SL and GOS distinctly modulate microbiota composition as shown by the outgrowth of *Bacteroides* and bifidobacteria species, respectively. Several of the abovementioned taxa belong to the class of Clostridia (i.e., *Rumincococcus* species), well-known producers of SCFAs, suggesting that supplementation of SL and (or) GOS in fecal batch cultures might alter SCFA production.

### SL and GOS Distinctly Modulate SCFA Production

**Figure 4:**
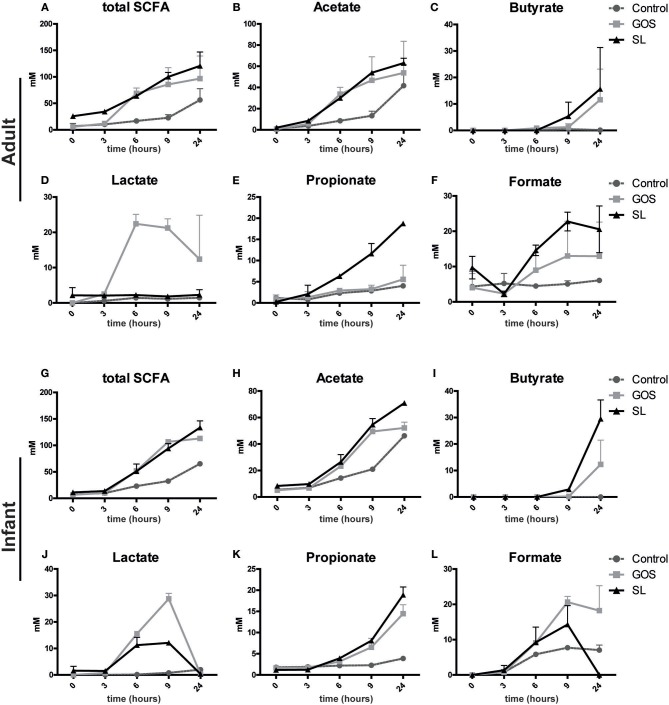
SL and GOS differentially stimulate SCFA production. Batch cultures of **(A–F)** adults and **(G–L)** infants pooled fecal samples were inoculated with SL or GOS. Fecal samples were collected at the start (0 h) and after a culture time of 3, 6, 9, and 24 h. SCFA levels were measured in the fecal samples by HPLC. SCFA levels of two independent batch cultures were represented as mean ± SEM.

Next, we assessed whether the microbial changes induced by SL or GOS altered SCFA production in the fecal batch cultures. SL and GOS both boosted the total production of SCFA (Figures 4A,G) including the production of acetate in adult- (Figure 4B) and infant batch cultures (Figure 4H). In adult batch cultures, GOS (Figure 4D) and SL (Figure 4E) induced the production of lactate and propionate, respectively. The concentration of lactate declined after 6–9 h of batch culturing, indicating that secondary producers may have used lactate as a carbon source (Figures 4D,J). This decline in lactate coincided with the increase of butyrate if growth medium was supplemented with GOS or SL (Figures 4C,I). These secondary producers include *Eubacterium halii* and *Faecalibacterium prausnitzii* and *Clostridium butyricum* as quantified in adult (Figure 3G) and infant (Figure 3H) batch cultures, respectively. Butyrate was also produced in batch cultures inoculated with SL in absence of lactate production (Figures 4C,D). In infant fecal cultures, lactate (Figure 4G) and propionate (Figure 4K) were produced in the presence of SL or GOS. The concentration of formate, acetate and total SCFA was higher in SL and GOS-containing cultures compared to batch cultures in baseline medium (Figures 4A,B,F,G,H,L). Thus, in line with the associated changes in microbiota composition, SL and GOS induce a distinct SCFA profile that is dominated by propionate and lactate, respectively.

## Discussion

Dietary fibers are widely considered to contribute to infant and adult intestinal health, via largely unexplored biological mechanisms that are thought to result from microbial fermentation of fibers into SCFAs. In this paper we show that SL and GOS both directly positively contribute to regulation of epithelial cell proliferation and differentiation *in vitro*. Transcriptome analysis of Caco-2 intestinal epithelial cells suggested that cell cycle pathways were modulated by SL and GOS; this could be corroborated in bioassays where phenotypes showing less proliferation and more differentiation were obtained. *In vitro* epithelial wound healing assays showed that SL and GOS also promoted re-epithelialization of a scratch area introduced in a confluent epithelium monolayer, an important aspect of wound healing. Additionally, our data showed that SL and GOS distinctly modulate microbiota composition, by promoting the outgrowth of *Bacteroides* and bifidobacteria, respectively. These changes in microbiota composition resulted in distinct changes in SCFA profiles, including increased propionate and lactate levels, respectively, and increased levels of butyrate for both SL and GOS.

The epithelial lining along the GI tract is the physical barrier that separates the intestinal lumen from the underlying tissue and protects against harmful antigens. Of note, gut closure (i.e., the state in which no macromolecules leak through the barrier) occurs in humans, in contrast to mice, already a few days after birth (15). Later in infancy, infection may temporarily disrupt barrier function, so continuous barrier function support is important to appropriately protect underlying tissues from invasion and to prevent inflammation. Nutritional intervention to support barrier function is especially relevant in premature infants who's gut function is characterized by immature mucosal and immune function and high level epithelial leakage, increasing the risk of uncontrolled inflammatory responses and the detrimental consequences of such response. Microbiota dysbiosis in combination with barrier dysfunction, and unbalanced immune responses can aggravate these detrimental consequences (16). HMO have been shown to improve intestinal homeostasis *in vitro* (56–58) and provide systemic anti-inflammatory effects in animal models (59).

We assessed the effect of GOS and SL on epithelial barrier functioning using *in vitro* assays investigating proliferation, differentiation and re-epithelialization. Our transcriptome analysis showed that GOS and SL both modulated the expression of genes involved in regulating stages of the cell cycle (e.g., cyclins and cyclin-dependent kinases; see Supplementary Text and Table S2). To investigate how this transcriptome profile showing modulation of pathways regulating cell cycle control translated into an epithelial phenotype in Caco-2 cells, we cultured the Caco-2 cells for 4 days in the presence of HMO. Cell counts after 4 days showed that at higher concentrations (>10 mg/ml) SL and GOS suppressed proliferation and induced differentiation, as shown by increased activity of alkaline phosphatase (ALP), a well-established differentiation marker for enterocytes. ALP was shown to modulate microbiota composition and inactivate different serotypes of LPS (60). The effects of SL that we observed are in line with previous findings by other groups that also reported suppressed proliferation and induced differentiation (57, 58). Thus, SL and GOS halt proliferation and induce production of ALP, which is a marker for intestinal homeostasis and epithelial differentiation. Importantly we show in this study that this effect was not restricted to SL, but was also observed when Caco-2 cells had been incubated for 4 days with GOS.

Since SL and GOS modulated very basic cellular mechanisms involved in epithelial differentiation, we questioned whether they are also capable of promoting re-epithelialization of epithelial cells, a process that involves tight regulation of proliferation and differentiation of epithelial cells. In the re-epithelialization phase of the “wound healing” process, cells migrate into the wound area, which involves reorganization of cellular cytoskeleton and cellular differentiation after the wound is closed (61, 62). Here, we observed that GOS and SL in concentrations present in cow's milk and breast milk can directly induce closure of the scratch area representing the wound in an *in vitro* model of wound healing. A well-known signaling cascade that induces re-epithelization is activation of the epidermal growth-factor receptor (EGFR) by ligands such as TGFα (63, 64). Interestingly, acidic HMO fraction and to a lesser extent, neutral HMO induced EGFR signaling in HT-29 cells (56), and we observed a similar boosting effect of the acidic SL and neutral GOS in our re-epithelialization model. Thus, we show here that oligosaccharides such as GOS and SL may promote epithelial barrier function and thus contribute to intestinal homeostasis. Future studies could investigate these effects on barrier function in early life using animal (e.g., allergy or colitis) models.

Since the early 1980's, research unraveled that breastfed infants show higher fecal numbers of bifidobacteria and a lower fecal pH compared to bottle-fed infants supplemented without prebiotics (65, 66). This lower pH of the stool in breastfed infants is caused by higher lactate and acetate levels (23, 67). Infant formula supplemented with a mixture of GOS/FOS promote a more bifidogenic microbiota composition (32–36) and a SCFA profile more similar to breast-fed infants (37, 67). In line with these findings, our batch cultures showed that GOS induced the outgrowth of bifidobacteria and increased production of lactate. Although to a lesser extent, SL also boosted the abundance of bifidobacteria, which is in line with earlier reports showing that different strains of bifidobacteria are capable of metabolizing both neutral and acidic HMO (7, 68, 69).

We showed that GOS induced the production of acetate, butyrate and lactate in fecal batch cultures of adults and, to a lesser extent, in infants. Animal models have shown that acetate can protect against *E.coli* infections in the gut (70) and systemic diseases such as asthma (26). The concentration of lactate declined rapidly after 9 h of the culture, indicating utilization by other bacteria which may include secondary butyrate producers (e.g., *Eubacterium halii and F. prausnitzii)* (71). Butyrate is known to induce anti-inflammatory response via binding G-protein coupled receptor (GPR) 109A and downregulation of NF-κB activity (72) and was shown to reduce diarrhea incidence by enhancing epithelial barrier functioning in piglets (73). As reviewed by others, acetate, butyrate and propionate also bind GPR43 and GPR41 and induce transcriptional and epigenetic modifications that results in anti-inflammatory effects. Hence, SCFA contribute to immune regulation and resolution of inflammatory diseases (e.g., allergy, and type I diabetes) (29). The degree of redundancy of the individual SCFA in eliciting these functions remains to be further investigated.

Multiple pathogenic bacteria and several commensal bacteria from different phyla encode genes that may participate in biochemical pathways that utilize sialic acid (74). Commensal bacteria that express pathways to utilize SL are *Bacteroides* members such as *B. fragilis, B ovatus* and *B. vulgatus*. Our data shows that *Bacteroides* increase in abundance upon culturing in the presence of SL. SL also increased the abundance of *Ruminococcus obeum* that was shown to switch to propionate production if fed with fucose or rhamnose (75). Propionate has been shown to exert systemic anti-inflammatory responses in allergic animal models for instance, suppression of allergic inflammation (27). Recent evidence shows that purified sialylated bovine milk oligosaccharides are mainly metabolized by *B. fragilis* (76), which is in line with *in vitro* cultures (77). The authors showed that sialylated oligosaccharides may be essential for optimal growth in early life. *B. fragilis* was also shown to contribute to immune homeostasis by producing polysaccharide A, a molecule that was shown to induce differentiation of CD4+ T-cells into regulatory T cells in the gut (78).

Importantly, our results show that SL and not GOS promotes the outgrowth of *F. prausnitizii* in adult batch cultures. GOS supplementation to elderly (79) or healthy adults did also not show a marked increase in fecal *F. prausnitizii* abundance (80). The infant pooled samples did not show the presence of *F. prausnitzii* at the start of the batch culture that might explain its absence after the incubation period with the inoculum (data not shown). Interestingly, at least some *F. prausnitzii* strains produce proteins that degrade sialic acid (74). *F. prausnitizii* induces anti-inflammatory responses which is shown to protect against colitis in animal models (81) and its prevalence has been reversely associated with the prevalence of ulcerative colitis in adults (82). However, increased *F. prausnitiziii* numbers were observed in pediatric Crohn's disease (83).

An important limitation of this study is the utilization of *in vitro* models using human epithelial cell lines and fecal batch cultures to study epithelial barrier functioning and microbiota composition, respectively. We fully acknowledge that the complex interactions between oligosaccharides, microbiota, metabolome, and host biology must be further studied using animal models and latest sequencing and -omics technologies. This study may initiate such future studies and allow for specific disease (e.g., colitis) models to be utilized.

## Conclusions

In conclusion, SL and GOS directly interact with the epithelial lining where they can support differentiation and wound repair. In addition, these compounds may distinctly modulate intestinal microbiota composition and activity and change the corresponding SCFA profiles in the gut lumen. The consequences of the effects in terms of the host health in infants and adults remain to be determined.

## Author Contributions

OP, EvdB, and MF-G conducted the experiments. OP, PvB, FS, and RvN analyzed the data. OP, PvB, MF-G, SB, HS, MK, and RvN contributed to interpretation of the results and writing process of the paper. All authors approve the final version of the paper.

### Conflict of Interest Statement

RvN is an employee of FrieslandCampina. The remaining authors declare that the research was conducted in the absence of any commercial or financial relationships that could be construed as a potential conflict of interest.
