# Corrigendum: Sialyllactose and Galactooligosaccharides Promote Epithelial Barrier Functioning and Distinctly Modulate Microbiota Composition and Short Chain Fatty Acid Production *In Vitro*

**DOI:** 10.3389/fimmu.2019.00762

**Published:** 2019-04-10

**Authors:** Olaf Perdijk, Peter van Baarlen, Marcela M. Fernandez-Gutierrez, Erik van den Brink, Frank H. J. Schuren, Sylvia Brugman, Huub F. J. Savelkoul, Michiel Kleerebezem, R. J. Joost van Neerven

**Affiliations:** ^1^Cell Biology and Immunology Group, Wageningen University & Research, Wageningen, Netherlands; ^2^Host-Microbe Interactomics Group, Wageningen University & Research, Wageningen, Netherlands; ^3^Microbiology and Systems Biology, The Netherlands Organization for Applied Scientific Research, Zeist, Netherlands; ^4^FrieslandCampina, Amersfoort, Netherlands

**Keywords:** epithelium, galactooligosaccharides, microbiota, short chain fatty acids, sialyllactose

In the original article, there was a mistake in Figure 3 as published. Figure 3C and Figure 3F were mistakenly swapped. The corrected Figure 3 appears below.

**Figure 3:**
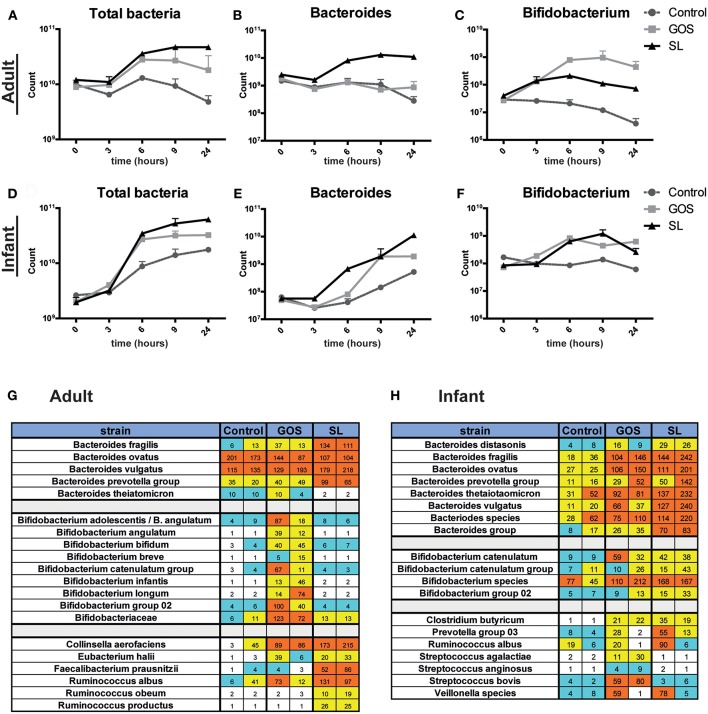
SL and GOS differentially modulate microbiota composition. Batch cultures of adult and infant pooled fecal samples cultured in growth medium were supplemented with or without SL or GOS in duplo. Fecal samples were collected at the start of the batch culture and after 3, 6, 9, and 24 h. Microbiota composition on genus level **(A–F)** and on species level **(G,H)** was determined by qPCR and chip analysis, respectively. Bacterial numbers were shown as mean ± SEM of two independent batch cultures. Raw fluorescence data are shown for both individual runs for chip analysis.

The authors apologize for this error and state that this does not change the scientific conclusions of the article in any way. The original article has been updated.

