# Supplementary material for: Sialyllactose and Galactooligosaccharides Promote Epithelial Barrier Functioning and Distinctly Modulate Microbiota Composition and Short Chain Fatty Acid Production In Vitro
Source: Front Immunol. 2019 Feb 12;10:94. doi: 10.3389/fimmu.2019.00094 (PMC6380229; doi:10.3389/fimmu.2019.00094)
Supplement: Supplementary file 1 [file Data_Sheet_1.docx]

**Supplementary text and tables S1 and S2: transcriptome data**

In this transcriptome study, we found, using Ingenuity Pathway Analysis (IPA), that cell cycle control stands out among the significantly modulated pathways. Intriguingly, our transcriptome analysis shows that GOS and SL modulate cell cycle control in a similar fashion in Caco-2 cells. Here, we provide a more extensive analysis of cell cycle genes that were significantly differentially regulated.

Cell cycle control is regulated by two key classes of molecules, cyclin-dependent kinases (CDKs) and their binding partners, the cyclins. In mammals there are a wide variety of CDK that form complexes with the corresponding stage-specific cyclins during different phases of the cell cycle (reviewed by Satyanarayana and Kaldis 2009). In the first phase of the cell cycle, the G1 phase of cell division, the cell increases in size and ensures it is ready for DNA synthesis. Cdk4 are activated by cyclin D proteins, resulting in the transcription of E2F transcription factors and cell cycle progression. E2F responsive genes are CCNA and CCNE, coding for cyclin A and E, respectively. Cyclin E activates Cdk2, resulting in E2F transcription, which results in entry into the S-phase (Satyanarayana & Kaldis 2009). Our transcriptome data shows that GOS and SL upregulate genes involved in G1/S phase progression; these genes can also be regulated by estrogen. This is in agreement with the predicted (IPA analysis) induction of the estrogen-mediated S-phase entry pathway by GOS. Our IPA output shows that this pathway is only induced by GOS and not by SL, which can be explained by the strict statistical cut-off value (i.e. FDR 3^-8^) that we used for the analysis of the transcriptome of SL stimulated Caco-2 cells. Nevertheless, SL also induced these genes involved in G1/S progression (Figure 2C).

At the beginning of the S-phase, cyclin A that phosphorylates proteins involved in DNA replication, is synthesized. Finally, cyclin B binds CDK1 to enter prophase and drive mitosis (Peters 2002). Our results showed that CDK1, CCNB1 and CCNB2 are upregulated by SL and GOS. Yet additional proteins regulate cell cycle. PLK activates CDC25 and together with CDK1 initiate the entry of G2 to mitosis phase. PLK and CDC25A and CDC25C are upregulated by SL and GOS. Cyclin B:CDK1 complexes are involved in the final stage of the cell cycle, inducing cell proliferation. PLK and MKLP1 are also needed for the formation of mitotic spindles (Sumara et al. 2004) and midzone formation (Zhu et al. 2005). In line with the upregulation of genes coding for the Cyclin B:CDK complex, these mitosis progression genes were upregulated as well by SL and GOS treatment. Proteins of the Cip/Kip family (e.g. p21^Cip1^ and p27^Kip1^) inhibit the activity of cyclin A, B and E activity thus blocking multiple stages of cell division. Interestingly, we found that p21^Cip1^, coded by CDK1NA had been 3 fold downregulated in SL and GOS treated Caco-2 cells. A table listing the differential expression of cell cycle control genes is provided below (Table S2). In summary, our IPA results showed that at least some concentrations of SL and GOS induce the transcription of genes that control multiple stages of cell division.

**Table S1. Number of pathways predicted to be induced or repressed by GOS or SL**

|  | GOS | SL |
| --- | --- | --- |
| total | 63 | 28 |
| NaN | 39 | 24 |
| positive Z-score | 8 | 1 |
| negative Z-score | 13 | 3 |
| Z-score = 0 | 3 | 0 |

**Table S2: Genes involved in cell cycle control as fold change compared to medium control. Genes were selected based on the SL dataset with a FDR cut-off value of 10^-8^.**

|  | **fold change** | |
| --- | --- | --- |
|  | **SL** | **GOS** |
| **AURKA** | **4.1** | **3.3** |
| **BLM** | **2.0** | **3.7** |
| **CCNA2** | **3.3** | **4.8** |
| **CCNB1** | **4.5** | **3.6** |
| **CCNB2** | **3.2** | **3.2** |
| **CCNE2** | **4.5** | **5.5** |
| **CDC25A** | **4.8** | **4.4** |
| **CDC25C** | **5.9** | **4.4** |
| **CDK1** | **6.5** | **6.5** |
| **CDKN1A** | **0.4** | **0.3** |
| **CHEK1** | **2.6** | **3.5** |
| **CKS2** | **7.6** | **4.4** |
| **CREB3L3** | **0.4** | **0.3** |
| **E2F1** | **5.6** | **3.8** |
| **E2F7** | **3.4** | **5.9** |
| **E2F8** | **3.9** | **5.2** |
| **FANCD2** | **0.5** | **4.2** |
| **FBXO5** | **4.0** | **3.7** |
| **HIPK2** | **2.9** | **3.1** |
| **KIF11** | **3.7** | **4.4** |
| **KIF23** | **7.3** | **5.5** |
| **MDM2** | **3.3** | **3.7** |
| **PCNA** | **3.0** | **3.8** |
| **PLK1** | **5.4** | **4.3** |
| **PLK4** | **6.1** | **4.9** |
| **PPM1D** | **6.6** | **2.7** |
| **PRC1** | **7.4** | **4.0** |
| **PTTG1** | **4.4** | **3.1** |
| **RPS6KA1** | **3.2** | **2.5** |
| **SFN** | **10.1** | **3.2** |
| **SMC2** | **3.7** | **3.8** |
| **SMC4** | **4.1** | **3.5** |
| **TOPB1** | **2.8** | **2.6** |
| **TP73** | **3.3** | **3.2** |
| **FOS** | **2.2** | **3.2** |
| **FOSL1** | **6.1** | **4.9** |

**References**

1. Satyanarayana A, Kaldis P. Mammalian cell-cycle regulation: Several cdks, numerous cyclins and diverse compensatory mechanisms. Oncogene (2009) 28:2925–2939. doi:10.1038/onc.2009.170

2. Peters J-M. The Anaphase-Promoting Complex. Mol Cell (2002) 9:931–943. doi:10.1016/S1097-2765(02)00540-3

3. Sumara I, N JFG-A, Gerlich D, Hirota T, Kraft C, Torre C de la, Ellenberg J, Peters J-M. Roles of Polo-like Kinase 1 in the Assembly of Functional Mitotic Spindles. Curr Biol (2004) 14:1712–1722. doi:10.1016/j

4. Zhu C, Bossy-Wetzel E, Jiang W. Recruitment of MKLP1 to the spindle midzone/midbody by INCENP is essential for midbody formation and completion of cytokinesis in human cells. Biochem J (2005) 389:373–381. doi:10.1042/BJ20050097
