# Supplementary figures and images for: Sialyllactose and Galactooligosaccharides Promote Epithelial Barrier Functioning and Distinctly Modulate Microbiota Composition and Short Chain Fatty Acid Production In Vitro
Source: Front Immunol. 2019 Feb 12;10:94. doi: 10.3389/fimmu.2019.00094 (PMC6380229; doi:10.3389/fimmu.2019.00094)

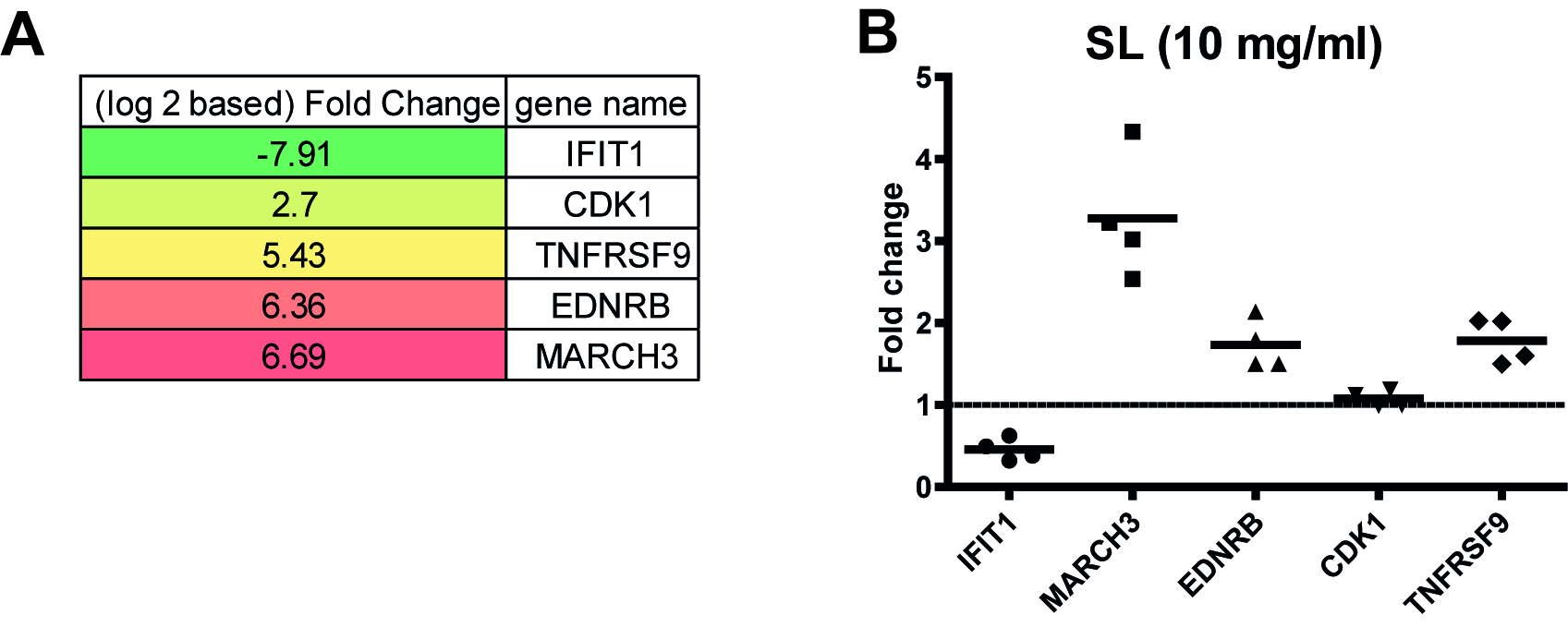

Supplement: Supplementary file 2 [file Image_1.TIF]

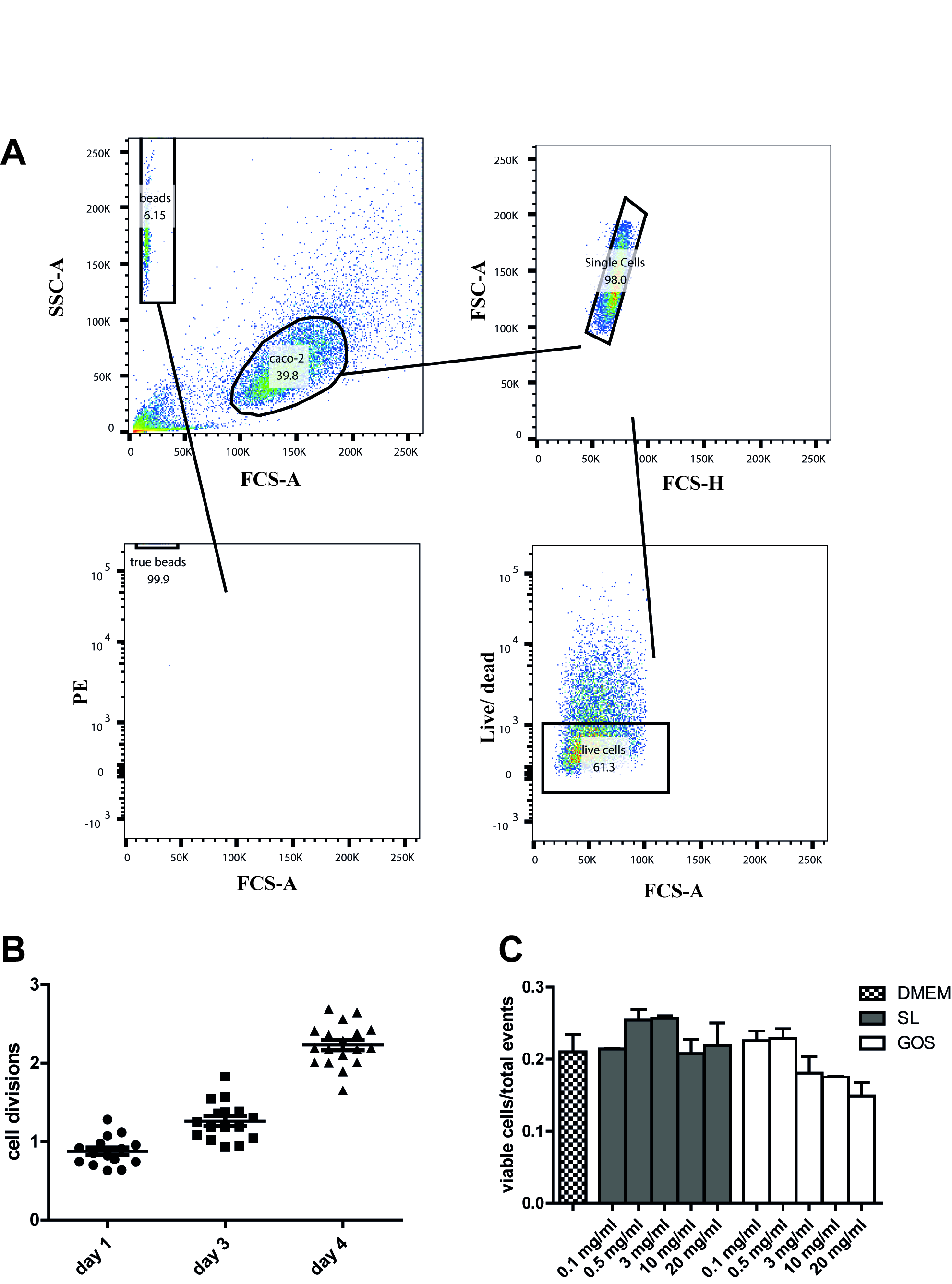

Supplement: Supplementary file 3 [file Image_2.TIF]

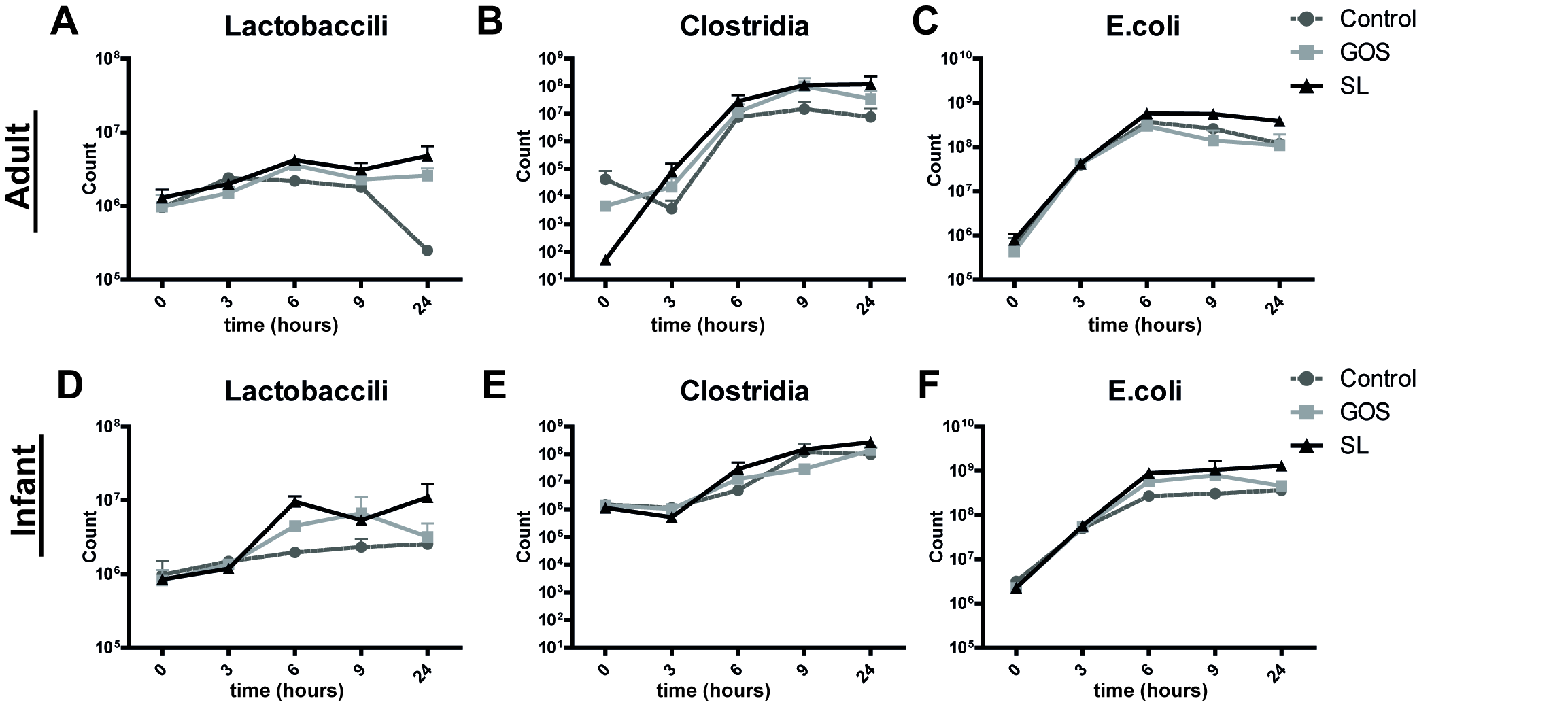

Supplement: Supplementary file 4 [file Image_3.TIF]
